# Organizing “Play Streets” during school vacations can increase physical activity and decrease sedentary time in children

**DOI:** 10.1186/s12966-015-0171-y

**Published:** 2015-02-13

**Authors:** Sara D’Haese, Delfien Van Dyck, Ilse De Bourdeaudhuij, Benedicte Deforche, Greet Cardon

**Affiliations:** Faculty of Medicine and Health Sciences, Department of Movement and Sports Sciences, Ghent University, Watersportlaan 2, 9000 Ghent, Belgium; Research Foundation Flanders (FWO), Egmontstraat 5, 1000 Brussels, Belgium; Department of Public Health, Ghent University, De Pintelaan 185, 9000 Ghent, Belgium

**Keywords:** Active play, Vacation, Children, Neighborhood, Intervention

## Abstract

A Play Street is a street that is reserved for children’s safe play for a specific period during school vacations. It was hypothesized that a Play Street near children’s home can increase their moderate- to vigorous-intensity physical activity (MVPA) and decrease their sedentary time. Therefore, the aim of this study was to investigate the effect of Play Streets on children’s MVPA and sedentary time.

A nonequivalent control group pretest-posttest design was used to determine the effects of Play Streets on children’s MVPA and sedentary time. Data were collected in Ghent during July and August 2013. The study sample consisted of 126 children (54 from Play streets, 72 from control streets). Children wore an accelerometer for 8 consecutive days and their parents fill out a questionnaire before and after the measurement period. During the intervention, streets were enclosed and reserved for children’s play. Four-level (neighborhood – household – child – time of measurement (no intervention or during intervention)) linear regression models were conducted in MLwiN to determine intervention effects.

Positive intervention effects were found for sedentary time (β = -0.76 ± 0.39; χ^2^ = 3.9; p = 0.05) and MVPA (β = 0.82 ± 0.43; χ^2^ = 3.6; p = 0.06). Between 14h00 and 19h00, MVPA from children living in Play Streets increased from 27 minutes during normal conditions to 36 minutes during the Play Street intervention, whereas control children’s MVPA decreased from 27 to 24 minutes. Sedentary time from children living in the Play Street decreased from 146 minutes during normal conditions to 138 minutes during the Play Street intervention, whereas control children’s sedentary time increased from 156 minutes to 165 minutes. The intervention effects on MVPA (β = -0.62 ± 0.25; χ^2^ = 6.3; p = 0.01) and sedentary time (β = 0.85 ± 0.0.33; χ^2^ = 6.6; p = 0.01) remained significant when the effects were investigated during the entire day, indicating that children did not compensate for their increased MVPA and decreased sedentary time, during the rest of the day.

Creating a safe play space near urban children’s home by the Play Street intervention is effective in increasing children’s MVPA and decreasing their sedentary time.

## Background

Physical activity is associated with numerous health benefits in children [1]. Besides, a recent meta-analysis showed that more sedentary time is related to negative health outcomes in 5- to 17-year-old children [2]. Therefore, children are encouraged to engage at least in 60 minutes of daily moderate- to vigorous-intensity physical activity (MVPA) [3] and to limit their sedentary time [4]. Despite the health benefits of engaging in sufficient physical activity and limiting sedentary time, many children do not meet the Physical activity guidelines and spend too much time sedentary [3].

Accessibility to screen-based activities, such as television, video games and computers/Internet, has largely increased, leading to higher levels of sedentary time in children [5]. Besides, children’s active outdoor play can significantly contribute to their MVPA [6,7], but compared to previous generations, children play less outside nowadays [8]. Safety concerns (e.g. road safety, stranger danger) may cause parents to restrict their children to play outdoors [9]. Therefore, interventions promoting children’s active outdoor play, by providing safe places to play outside during leisure time are important and can be effective in increasing their overall physical activity and decreasing their sedentary time.

Traditionally, interventions promoting physical activity focused on changing personal and psychosocial factors (e.g. knowledge of health benefits). However, these interventions have demonstrated limited success in promoting long-term maintenance of health behaviors [10]. Furthermore, by focusing on changing personal and psychosocial factors, only small groups of people can be reached, whereas intervening in the environment could have positive outcomes on larger groups of people living in that environment. Consequently, ecological models of health behavior have gained increased attention. From an ecological perspective, physical activity and sedentary time are not only influenced by individual factors, but also by the social (e.g. family) and physical (e.g. neighborhood) environment [11]. Ecological models emphasize the interaction between the individual and factors at multiple levels (e.g. social factors and environmental factors) [11]. Therefore interventions at the neighborhood level may help to increase MVPA and decrease sedentary time in large groups of children.

However, studies evaluating neighborhood interventions to increase children’s physical activity and decrease their sedentary time are scarce. An Australian study investigated the effect of park improvements on park activity [12]. Park improvements (including the permanent establishment of a walking track, a barbecue area and a playground, a fenced leash-free area for dogs, landscaping and fencing to prevent motor vehicle access to the park) were positively associated with the number of children visiting the park and the number of people observed walking and being vigorously active [12]. Also in the US, park renovations (i.e. the renovations of the playfields that were primarily used for soccer and baseball) increased visitation and overall physical activity in different age groups [13]. Furthermore, a US study evaluated a neighborhood intervention to increase children’s physical activity. Schoolyards were made available on week- and weekend days as a safe play space for children. This led to an increase in children’s physical activity and a decrease in watching television, movies and DVDs and playing video games on weekdays [14]. Another US study, examining the impact of renovations of schoolyards available outside school hours on children’s physical activity showed that children were more active at schools with renovated schoolyards compared to schoolyards in control schools [15,16]. However due to the restricted independent mobility of children [17], it seems that these kind of interventions are probably only effective for children who live near a park or a school.

An intervention that can be implemented in most residential streets and neighborhoods, is the so called “Play Street” intervention. Since 1998, Play Streets are organized in different Belgian cities and villages during school vacations and are a collaboration between the inhabitants of the Play Street and the city council. A Play Street is a street that is reserved for children’s safe play for a specific period during school vacations. In Play Streets organized in Belgium, motorized traffic is generally prohibited and only local traffic is allowed at a footpace. Children playing in the Play Street may not be hindered or endangered. The rules and timing of the Play Street are determined by the city council and may differ across different cities and villages.

Play Streets offer children a safe play space to be active in their own neighborhood. By organizing Play Streets, the neighborhood environment (e.g. creating a car-free play place) and the social environment (e.g. increased social interaction between children playing in the street) are targeted. However, the effect of Play Streets on children’s MVPA and sedentary time has not been investigated so far. Therefore, the aim of this pilot study was to test the effectiveness of Play Streets to increase urban children’s MVPA and to decrease their sedentary time.

## Methods

### Procedure

A list with all Play Streets in Ghent (Flanders) during summer vacation (2 months: July and August) 2013 (n = 79) was obtained from the city council. In total, 16 Play Streets were organized during the weekends (on both Saturdays and Sundays), 13 Play Streets were organized only on Saturdays, 22 were organized only on Sundays, 22 Streets were organized for at least one week (i.e. 7 consecutive days) and 6 Play Streets were organized on another day of the week (3 on Wednesdays, 2 on Fridays and 1 on Mondays). Nineteen Play Street projects that lasted at least 7 consecutive days were selected.

Ghent consists of 201 statistical sectors, these are the smallest administrative entities (average area = 0.786 km^2^ ± 0.990) for which statistical data, are available. For each Play Street, a control neighborhood (i.e. statistical sector) with comparable walkability [18] characteristics and annual household income (National Institute of Statistics–Belgium, 2008) in Ghent was selected. The distance between the Play Streets and the control neighborhoods was on average 4.9 ± 3.4 km. In Play Streets and in adjacent streets that were directly connected to the Play Street, and in comparable control neighborhoods, door-to-door visits were conducted and in total 209 children (89 from Play Streets) were reached (305 hours of recruitment) who met following 2 inclusion criteria: 1) being in primary school or starting primary school after summer school vacation or finished primary school in June (age 6-12) and 2) residing at home during the one week measurement period (not going on holidays, not staying with friends or grandparents,…). From these 209 children, 167 (response rate = 79.9%) children (71 from Play Streets) obtained written informed consent from their parents to participate in the study.

**Figure 1:**
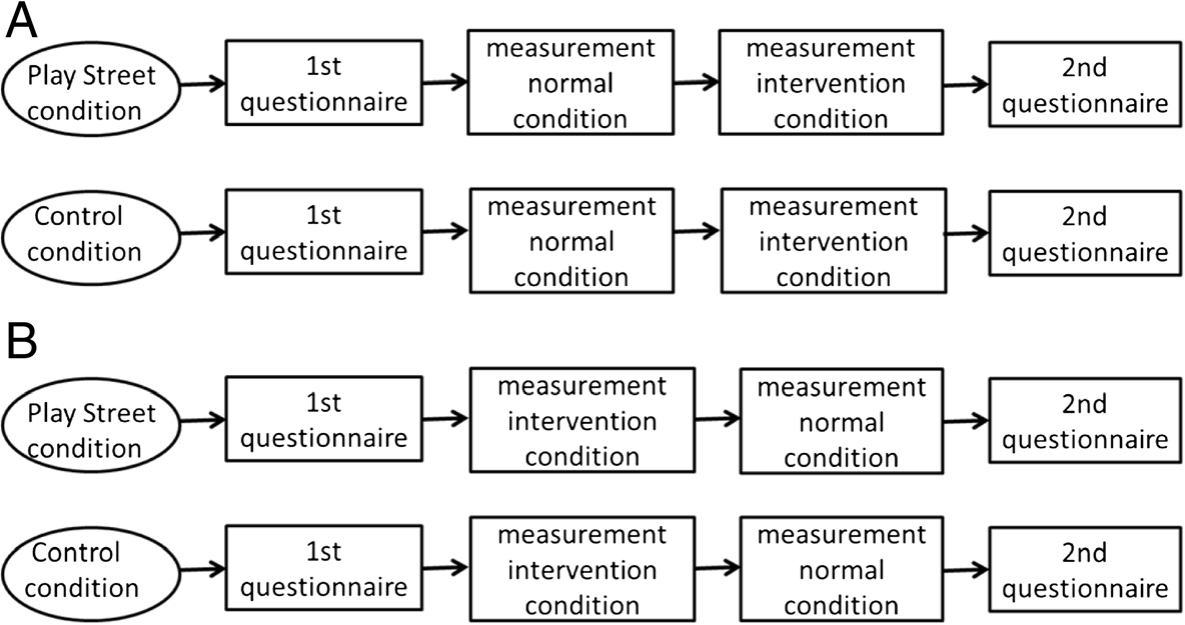
**Outline of the study design.** **A**: children first measured during normal condition and afterwards during intervention condition. **B**: children first measured during intervention condition and afterwards during normal condition.

A nonequivalent control group pretest-posttest design was used in the present study with the ‘pre’test (=under normal conditions) occurring during a normal week and the ‘post’test (=during intervention condition) occurring during the Play Street week (Figure 1). Children were asked to wear an accelerometer for 8 days (half a week during a normal week and half a week during a Play Street week) and parents were asked to complete a questionnaire concerning demographic variables before the measurement week (questionnaire 1) and to complete a questionnaire concerning Play Streets after the measurement week (questionnaire 2). Questionnaire 2 was different for parents from control streets and from intervention streets. The data collection was counterbalanced. In half of the intervention streets children were measured first during normal conditions and afterwards during the intervention condition (A in Figure 1), whereas in the other half of the intervention streets children were measured during the intervention condition first and afterwards during normal conditions (B in Figure 1). In control streets, measurements were performed at the same time as in their comparable Play Street. All Play Streets included in the present study started on a Monday and lasted at least one week. The Ethics Committee of the Ghent University Hospital approved the study.

### Play Street intervention

As mentioned in the introduction, Play Streets are car-free play places for children that are organized in different streets in cities and villages in Belgium. Characteristics that were described in the introduction apply to all Play Streets that are organized in Belgium. In the following part, characteristics that apply specifically to Play Streets in Ghent are described. The Play Streets in the current study were all organized in Ghent (237000 inhabitants, 15685 km^2^). A street in Ghent is eligible to become a Play Street if the street complies with the following conditions: the street is a residential street, with a speed limit of maximum 50 km/h, there is no significant passing traffic (e.g. public transport, no firehouse in the street), and the surrounding streets remain accessible after the introduction of the Play Street. If the street meets all these requirements, every street inhabitant can fill out an application for a Play Street at the city council. The application is for one period only. Thus in case the inhabitants want a Play Street again the year after, the application needs to be renewed. When the city council approves the application, the majority of the households in the street has to agree with the approved application. Besides, at least 3 volunteers living in the street have to sign an agreement with the city council to hold responsibility for the organization of the Play Streets. They are the contact persons between the city council and the other street inhabitants. The task of the volunteers is to inform the street inhabitants about the rules and timing of the Play Streets. Each day the Play Street is organized, the volunteers enclose the Play Street with fences and a traffic sign, indicating that car traffic is forbidden in the streets. The fences and traffic signs are delivered by the city council. When the Play Street is not used on a specific day (e.g. due to heavy rain), the Play Street can be cancelled by the volunteers on that day. Parents remain responsible for their children playing in the street. The city council regulates insurance for the volunteers.

In Ghent, a Play Street is organized between 14h00 and 19h00. The city council of Ghent also offers a box with play equipment that can be hired for free by the volunteers of the Play Streets. They can keep the box during the period of the Play Street intervention. The box includes balloons, water balloons, flags, chalks, rackets, balls, etc. When the Play Street ends, the volunteers have to return the box to the city council. In addition, other play materials are available such as popular games, a trampoline, a bouncy castle, a circus box etc. that can be hired for free for 1 day. The intervention was mainly developed to encourage free play. However, inhabitants were free to organize activities themselves (e.g. a barbeque, a sports afternoon,..). The volunteers could also apply for 1 organized activity during the Play Street intervention that is organized by the city council. This organized activity is the visit of a circus school that teaches circus skills to the children. After the intervention, the city council askes the volunteers of the Play Streets to fill out an evaluation form concerning the Play Streets.

In Ghent, a street can become a Play Street for maximally 14 days in July and August (e.g. every Sunday, every weekend in July, one week in July and 1 week in August, 14 consecutive days). This limit was set by the city council to reduce nuisance for the inhabitants of the play street and adjacent streets without children.

### Measurements

#### Demographic variables

Children’s age and sex were derived from the questionnaire 1. Parents were asked to report their own and their partner’s level of education. Educational attainment was used as a proxy for family socio-economic status (=SES). Families were classified as high SES-families if at least one parent had a college or university education; otherwise they were classified as low SES families. When parents did not fill out the question about their educational attainment (n = 14; 2 from Play Streets)), the dichotomized neighborhood income score was used as a proxy for family SES. Average daily temperature and rainfall were obtained from “www.weerstationoverijse.be”.

#### Play Street questionnaire

In questionnaire 2, parents’ opinions about Play Streets were assessed both in control and intervention streets. An outline of these questions is given in Table 1.

**Table 1:** **Outline of the questionnaires concerning the Play Streets in intervention streets and control streets**

	**Number of respondents**	**Totally disagree**	**Rather disagree**	**Neutral**	**Rather agree**	**Totally agree**
	**n**	**(%)**	**(%)**	**(%)**	**(%)**	**(%)**
**Play Street questionnaire 2°:**						
My child was enthusiastic about the Play Street.*	32	0.0	0.0	6.3	18.8	75.0
My child had a lot of friends in the Play Street.*	32	3.1	0.0	18.8	31.3	46.9
It was safe to play in the Play Street for my child.*	32	3.1	18.8	6.3	25	46.9
Thanks to the Play Street intervention, I had more social contact with neighbors.*	32	9.4	3.1	28.1	25.0	34.4
I had the impression that my child played more outside during the Play Street intervention as usual.*	32	15.6	9.4	12.5	18.8	40.6
The presence of the box with play equipment was valuable.**	21	9.5	9.5	42.9	4.8	33.3
**Control street questionnaire 2°°:**						
If there would be a play street in my neighborhood, children from my area would have more social contact with each other.	37	2.6	7.9	13.2	34.2	42.1
If there would be a play street in my neighborhood, adults from my area would have more social contact with each other.	38	0.0	13.2	23.7	42.1	21.1
In our neighborhood, there is insufficient play space for the children.	38	26.3	26.3	23.7	10.5	13.2

*answers from parents whose children used the Play Street.

**answers form parents in a Play Streets with the box available.

°the questionnaire that parents from children in Play Streets, filled out after the measurement period.

°°the questionnaire that parents from children in control streets, filled out after the measurement period.

In Play Streets, parents were also asked to indicate if they lived in the Play Street or in a street nearby and if their child did use the Play Street. Parents also ticked in which of the following activities their child took part during the intervention: active games (e.g. tag), board games, reading, ball games (e.g. soccer, basketball), cycling, organized activities or other activities. Parents from children in intervention streets were asked ‘did your child use the Play Street in your neighborhood’ and if yes; how many times their child used the Play Street. Response options were: daily, every weekend day, every weekday, during the week but not daily, during the weekend but not daily, 1 day per week.

In control streets, parents were asked to indicate if their child would participate in the Play Street if they lived nearby a Play Street (yes-maybe-no).

#### Physical activity

Objective physical activity between 14h00 and 19h00 and during the entire day was determined by accelerometers. The specific period between 14h00 and 19h00 was studied, as the intervention took place from 14h00 until 19h00. Children wore an Actigraph™ GT1M, GT3X or GT3X+ accelerometer (15 s epoch) during waking hours for 8 consecutive days starting on Wednesday. Strong agreement was found between these three activity monitors for measuring MVPA in children, making it acceptable to use different models within a given study [19].

The accelerometer was worn on the right hip with an elastic waist belt. Accelerometer data were screened, cleaned and scored using data-reduction software MeterPlus 4.2. Periods of 20 minutes of consecutive zeros or more were removed and defined as non-wear time. MVPA and sedentary time were calculated using the cutpoints of Evenson [20], during the intervention period (14 h – 19 h) and during the entire day. These cutpoints were recommended in a comparative validity study of accelerometer cutpoints [21]. Only children who had data on at least 1 day in the intervention condition or 1 day under normal conditions were included in the analyses. For the analyses concerning the intervention period (14h00 – 19h00), a valid day was defined as a day with minimum 2.5 h wearing time, for the analyses concerning the entire day, a valid day was defined as a day with at least 8 hours of wearing time.

### Analyses

SPSS 20 was used to describe the characteristics of the sample. To describe average minutes of MVPA and sedentary time from 14h00 until 19h00, the following formula was used: (average MVPA/average wear time)*300. This formula was used to take into account the different wear times. Four-level (neighborhood – household - child – time of measurement (no intervention or during intervention)) linear regression analyses with random intercept and fixed slopes were conducted in MLwiN 2.25 to investigate possible intervention effects [22]. Intervention effects were examined on MVPA and sedentary time from 14h00 until 19h00 and during the entire day. Analyses were controlled for age, sex, family SES, average temperature (°C), average rainfall (l/m^2^), the number of valid days and valid wear time. The IGLS (Iterative Generalised Least Squares) estimation method in MLwiN was used to conduct the multilevel regression analyses. Physical activity and sedentary time variables were square root transformed to obtain normality before entering them into the regression analyses. The β-value for the interaction between ‘time’ and ‘condition’ was used to investigate whether differences in MVPA and sedentary time between a normal week and an intervention week differed between children in the intervention and normal condition. P-values ≤ 0.1 were considered as significant for the interaction terms. Higher significance levels are used for interaction terms as they have less power [23].

## Results

### Descriptive results

In total, 126 children had valid accelerometer data (72 from control streets and 54 from Play Streets) and were included in the analyses. Of the total sample, 54.8% were boys, 37.3% had low family SES and the mean age was 9.0 ± 2.1 years.

#### Intervention group

Of the intervention group, 59.3% were boys, 38.9% had low family SES and the mean age was 8.7 ± 2.2 years. In the intervention group, 81% of the children lived in the Play Street, whereas 19% of the children lived nearby the Play Street. Of all children, 80.5% played in the Play Street during the intervention. Of the parents whose child played in a Play Street (80.5%), 62.5% reported daily use of the Play Street, 6.3% used the Play Street every weekday, 15.6% used the Play Street during the week but not daily, 15.6% used it 1 day per week. Parents whose children played in the Play Street indicated that their children mainly engaged in active games (e.g. tag) (78.1%), ball games (61.3%) or cycling (67.7%) and less in organized activities (25.8%), board games (6.5%) or reading (3.2%). Of the children playing in the streets, 67.8% had access to the hired box with play equipment and 25.0% of the parents indicated that this box was valuable. From the parents whose children played in the Play Street, 93.8% agreed that their children were enthusiastic about the Play Street, 78.2% agreed that their children had a lot of friends in the Play Street and 71.9% agreed that it was safe to play in the Play Street. According to 59.4% of the parents, there was more social contact with neighbors during the Play Street week and 59.4 % of the parents had the impression that children played more outside in the Play Street compared to a normal week. Volunteers of 12 streets in the current sample filled out the evaluation form of the city council; 7 of them hired the box with play equipment and 7 of them hired other play materials (e.g. a bouncy castle, a trampoline) (Table 1).

#### Control group

Of the control group, 51.4% were boys, 36.1% had low family SES and mean age was 9.3 ± 2.0 years. In the control group, 89.2% of the parents indicated that their children would play in the Play Street, if there was a Play Street in their neighborhood. In the control streets, the parents agreed that children (76.3%) and adults (63.2%) would have more social contact with other children and adults if there would be a Play Street in their neighborhood; 23.7% of the parents agreed that there is insufficient play space for their children in their neighborhood (Table 1).

### Effect of the Play Street intervention on sedentary time and MVPA between 14h00 until 19h00

Intervention effects of Play Streets from 14h00 until 19h00 are described in Table 2. Significant differences in sedentary time (β = -0.76 ± 0.39; χ^2^ = 3.9; p = 0.05) and MVPA (β = 0.82 ± 0.43; χ^2^ = 3.6; p = 0.06) were found between a normal week and an intervention week depending on the condition (control street or intervention street). In control streets, sedentary time was higher (164.6 mins/day vs. 156.5 mins/day) and MVPA was lower (24.3 mins/day vs. 26.9 mins/day) during the intervention condition compared to the normal condition, whereas in intervention streets, sedentary time was lower (137.7 mins/day vs. 146.3 mins/day) and MVPA was higher during the intervention condition (35.8 mins/day vs. 26.7 lubs/day).

**Table 2:** **Intervention effects on sedentary time and moderate- to vigorous-intensity physical activity (MVPA)**

	**Period**	**Condition**	**n**	**Average normal condition**	**Average intervention condition**	Time * Condition ^**a**^	**Χ** ^**2**^	**p**
				**Min/day (SD)**	**Min/day (SD)**	**Β (SE)**		
**Sedentary time** ^**b**^	14 h – 19 h	Control street^c^	72	156.49 (41.69)	164.61 (40.10)	−0.759 (0.385)	3.896	0.048
		Intervention street^i^	54	146.30 (38.36)	137.74 (35.43)			
**MVPA** ^**b**^	14 h – 19 h	Control street^c^	72	26.91 (16.92)	24.32 (13.47)	0.816 (0.428)	3.626	0.057
		Intervention street^i^	54	26.70 (13.51)	35.79 (24.93)			
**Sedentary time** ^**b**^	Entire day	Control street^c^	71	381.50 (92.39)	400.33 (94.43)	−0.616 (0.246)	6.301	0.012
		Intervention street^i^	51	367.46 (110.72)	336.69 (72.92)			
**MVPA** ^**b**^	Entire day	Control street^c^	71	57.41 (33.68)	52.87 (27.98)	0.854 (0.332)	6.623	0.010
		Intervention street^i^	51	54.92 (24.94)	67.05 (38.00)			

^a^ = adjusted for age, sex, family SES, average daily temperature, average daily rainfall, number of valid wear days and accelerometer wear time.

^b^ = square root transformed.

SD = standard deviation.

SE = standard error.

Χ^2^ = chi square.

MVPA = moderate- to vigorous-intensity physical activity.

n = number of children in the analyses.

^c^ = streets having no Play Street.

^i^ = streets having a Play Street.

### Effect of the Play Street intervention on sedentary time and MVPA during the entire day

Intervention effects of Play Streets during the entire day are described in Table 2. Significant differences in total daily sedentary time (β = -0.62 ± 0.25; χ^2^ = 6.3; p = 0.01) and total daily MVPA (β = 0.85 ± 0.33; χ^2^ = 6.6; p = 0.01) were found between a normal week and a Play Street week depending on the condition (control street or intervention street). In control streets, sedentary time was higher (400.3 mins/day vs. 381.5 mins/day) and MVPA was lower (52.9 mins/day vs. 57.4 mins/day) during intervention condition compared to the normal condition, whereas in intervention streets, sedentary time was lower (336.7 mins/day vs. 367.5 mins/day) and MVPA was higher during intervention condition (67.1 mins/day vs. 54.9 mins/day).

## Discussion

This study was the first to investigate the effect of Play Streets on children’s physical activity and sedentary time. Positive intervention effects were found for MVPA and sedentary time between 14h00 and 19h00, and during the entire day. Between 14h00 and 19h00 children’s MVPA in the Play Streets group increased from 27 minutes/day during normal conditions to 36 minutes/day during the Play Street intervention, whereas MVPA in the control children decreased from 27 to 24 minutes/day. During the entire day, children’s MVPA in the Play Streets group increased from 55 minutes/day to 67 minutes/day, whereas MVPA in the control children decreased from 57 minutes/day to 53 minutes/day. This indicates that children did not compensate for their increased MVPA during the intervention period during the rest of the day, as they engaged in more MVPA during the entire intervention day. The fact that children did not compensate for their increased PA during intervention time was also found in different other studies [24,25]. However, these findings are in contrast with findings from Fremeaux et al., who stated that more activity at one time (e.g. during the intervention) will be compensated for by less activity at another time (e.g. in the morning) [26] due to the effect of intrinsic biological control on physical activity [27]. However, in another Belgian study investigating the effects of lowering playground density during recess, it was also found that boys did not compensate more MVPA during recess at another moment during the school day. However, girls who engaged in more MVPA during recess, compensated for this increase by decreasing their MVPA during another moment during the school day [28]. It is possible that in the current study, differences between boys and girls also existed, however due to the small sample size it was not possible to investigate the intervention effects in boys and girls separately.

In children from the intervention group, MVPA during the intervention period contributed more to the entire day Physical activity during intervention condition (53.4%) compared to the normal condition (48.6%). Although changes in MVPA were relatively small, Play Streets can positively contribute to obtain the physical activity guidelines of 60 minutes MVPA/day. On a weekly basis, children will engage in 63 minutes MVPA more during the Play Street intervention compared to a normal week. When children had a Play Street available, they engaged in MVPA in 11.9% of the time, whereas children in control streets engaged in MVPA for 8.1% of the time during the same time period. Parents from children using the Play Street indicated that their child mainly participated in active games, ball games and cycling in the Play Street, whereas only a small part of the children engaged in board games or reading in the Play Street. This engagement in active games can explain the increase in MVPA during the intervention condition. Only 25.0% of the parents agreed that the box with play equipment was valuable for the Play Street. Possible reasons are the fact that manuals or rules were lacking or that the play material was incomplete. Furthermore, it is possible that children in the Play Street mainly engaged in activities that did not require play equipment but engaged in activities requiring space (e.g. 67.7% of the children engaged in cycling) and interaction with other children (e.g. 78.1% of the children engaged in active games and 61.3% in ball games). Larger effects on MVPA could possibly have been found if the box with play material contained attractive play equipment to be physically active.

Between 14h00 and 19h00, children’s sedentary time in the Play Street group decreased from 146 minutes/day during normal conditions to 138 minutes/day during the Play Street intervention, whereas sedentary time in the control children increased from 156 minutes to 165 minutes/day. During the entire day, sedentary time in de Play Street group decreased from 367 to 337 minutes/day, whereas sedentary time in the control children increased from 382 to 400 minutes/day. This indicates that the Play Street can contribute to lowering sedentary time and that a Play Street can be a nice and amusing alternative for screen-based activities, such as television viewing, video games and computer/Internet. However, children’s levels of sedentary behavior were still quite high when their street turned into a Play Street. This indicates that the Play Street intervention needs to be combined with other efforts to decrease sedentary time. The decrease in MVPA and increase in sedentary time in children in control streets are possibly due to external factors (e.g. the absence of other children during the intervention condition).

Given the fact that during the summer vacation (July and August), the risk for weight gain in children is high [29], and more MVPA [1] and less sedentary time [2] are related to a more healthy weight status, a Play Street intervention in combination with other strategies and organized over a longer period to increase MVPA and decrease sedentary time can be helpful in the prevention of children’s overweight and obesity. For example, it is also possible that Play Streets can have a stronger effect on MVPA and sedentary behavior if more organized activities will take place in the Play Street. For example, a sports teacher that organizes some games for the children (e.g. parachute games, a sports event,..) might be an effective strategy to further increase children’s MVPA. Also the content of the box with play equipment could be adapted with more material to increase MVPA and to decrease sedentary behavior (e.g. exclusion of chalks). Furthermore while the maximum length of the Play Streets in Ghent is currently only two weeks, it might be preferable to implement this intervention for a longer period (eg entire summer vacation). In Belgium, most recreational sports clubs are closed during summer vacation. Therefore, Play Streets can be an appropriate alternative to be active during summer vacation. Furthermore, it might be valuable to further organize Play Streets during the school year on Wednesday afternoons or on weekend days, as Belgian schools do not run on Wednesday afternoon and their physical activity levels are usually lower on weekend days compared to weekdays [30]. Further efforts are also needed to reach children from adjacent streets to play in the Play Streets, as these children were not always aware of the Play Streets in the neighborhood.

In Belgium, similar as in other countries, sports camps are organized during summer vacation. However, children from low SES families cannot always afford to let their child participate in a sports camp (e.g. due to the high costs) [31]. The Play Street intervention is an effective intervention that is free of costs for the inhabitants and therefore Play Streets may be especially valuable in low income neighborhoods.

The introduction of a Play Street offers a safe play space for children, away from traffic, nearby their home. Therefore, Play Streets can be an alternative for physical activity in the garden, in the park or in a playground, as safety concerns (e.g. road safety, stranger danger) cause parents to restrict their children to play outdoors [9]. The garden, parks and playgrounds were frequently reported places for active play in an Australian study [32]. However, a disadvantage of using parks or playgrounds is the fact that, especially in younger children, parents need to accompany their child to parks or playgrounds due to children’s restricted independent mobility. As Play Streets are located nearby children’s home, less parental supervision is necessary to let children play in Play Streets. A Play Street can be especially valuable for children without a garden at home. In Australian studies it was shown that children were mostly active outdoors in their garden [32,33]. A Play Street can be seen as a garden or park that is created near home. Another advantage of Play Streets is the presence of ‘passive supervision’ from parents in the street. Local parents can keep an eye on children playing in the street, which may lead to feelings of safety in children and their parents. This passive supervision is not present in parks or public playgrounds. About 60% of the parents from children that played Play Streets had the impression that their child played more outside during the intervention. Play Streets seem to be inviting for children to play outside.

Besides the beneficial effect on children’s physical activity and their sedentary time, it is also possible that Play Streets have beneficial effects on the social interaction in the neighborhood. About 60% of the parents indicated that they had more social contact with their neighbors during the Play Street intervention and more than 75% of the parents agreed that their child had a lot of friends in the play street. Most of the children played in games requiring social interaction (e.g. ball games), which may lead to more friends in the neighborhood. So, a Play Street can possibly be beneficial for children’s physical activity in two ways. First, there is possibly a direct effect of the provision of a safe play space for children on their physical activity. Secondly, the Play Streets can lead to increased contact between children and adults, which in turn can lead to increased physical activity in children, as the presence of a social network and friends in the neighborhood have been identified as important correlates of children’s physical activity [32]. However, future research should focus on the effects of Play Streets on social interaction in the neighborhood into more detail to reveal if the introduction of Play Streets can lead to more interactions and increased trust between adults and children and if these social interactions and trust also lead to more physical activity in children.

The effects of this intervention should also be investigated in different age groups (including preschoolers and toddlers), and for different time periods (e.g. the effect of a Play Street that is organized every Sunday). Further research should also investigate the intervention effects when a Play Street is implemented over a longer time period and if the intervention can lead to maintained behaviors over a longer time period. Furthermore, this intervention may be more effective in low versus high income neighborhoods, because Play Streets can be an alternative for expensive sports camps that are organized during summer vacations [31]. However, due to the small sample size it was not possible to investigate the intervention effects for different sub groups (e.g. high versus low SES groups, boys versus girls) separately. This forms a limitation of the study. Another weakness is the short measurement period of the accelerometers (i.e. 8 consecutive days) and the low requirements for number of valid days. The requirements for inclusion concerning the number of valid days were kept low in order to retain sufficient power in the analyses. To overcome this weakness, we controlled our analyses for number of valid days and valid wear time. A strength of the study is the use of accelerometers as objective measurement tools for physical activity and sedentary time. Moreover, the Play Street intervention was a sustainable and easy-implementable intervention. A Play Street is a very flexible intervention at neighborhood level that is successful due to the willingness of the parents to be involved in the intervention. The Play Street intervention than can be implemented in different streets depending on the need of a Play Street in specific streets.

## Conclusions

The introduction of a Play Street, leading to a safe play space near home, is an effective intervention at neighborhood level to increase urban children’s MVPA and decrease their sedentary time during summer vacations. Therefore, Play Streets can be a valuable alternative for children without a garden, or can be an alternative for parks that are usually further away from children’s home. However, additional interventions aiming to increase MVPA and to decrease sedentary time are still necessary in order for children to reach the health guidelines for MVPA and sedentary time. More research is necessary in order to reveal the effect of Play Streets in different age groups and sub groups and for different time periods.

## Abbreviations

MVPA: Moderate- to vigorous-intensity physical activity
SES: Socio-economic status
